# Family Influences on the Dental Caries Status of Children with Special Health Care Needs: A Systematic Review

**DOI:** 10.3390/children9121855

**Published:** 2022-11-29

**Authors:** Diyana Shereen Anwar, Mohd Yusmiaidil Putera Mohd Yusof, Mas Suryalis Ahmad, Budi Aslinie Md Sabri

**Affiliations:** 1 Centre of Population Oral Health and Clinical Prevention Studies, Faculty of Dentistry, Universiti Teknologi MARA, Sungai Buloh 47000, Selangor, Malaysia; 2 Institute of Pathology, Laboratory and Forensic Medicine (I-PPerForM), Universiti Teknologi MARA, Sungai Buloh 47000, Selangor, Malaysia; 3 Centre of Special Care Dentistry, Faculty of Dentistry, Universiti Teknologi MARA, Sungai Buloh 47000, Selangor, Malaysia

**Keywords:** family influences, dental caries, children with special health care needs, systematic review

## Abstract

Oral health is a likely source of health inequalities in children with special health care needs (CSHCN), according to multiple studies. This systematic review aimed to explore the evidence of family influence—as well as family features, such as societal variables and parents’ perspectives—on these children’s dental caries status. Embase, Ebsco, Scopus, PubMed, and Web of Science databases were all searched. All cross-sectional research published on the variables of family impact ranging from 2010 to 2021 were considered. The DMFT and dmft index was utilised for the report data of dental caries, whereas the National Heart, Lung, and Blood Institute (NHLBI) was used for the purposes of assessing quality. Of the 3861 records identified, 14 were eligible. Further, most of the studies had a caries prevalence of over 50%, (*p*-value < 0.005). Family influences imparted a significant relationship and association towards the dental caries status of the demographic being studied. The influences on the children included tooth brushing behaviour, parents’ education level, parents’ occupation level, diet pattern, dental visit, snacking frequency, parents’ psychological status, family size, and parents’ knowledge towards oral health. Future research should further explore the family functioning domains, specifically among the CSHCN population. This study was registered on PROSPERO, number CRD42021274923.

## 1. Background

The World Health Organization (WHO) has defined disability as: (1) impairment, this can be in regard to body structure or mental functioning; (2) activity limitation, such as difficulty in walking, seeing, or hearing; and (3) participation restrictions faced by a person, where this can be, for example, in normal daily activities, such as working, engaging in social and recreational activities, or, also, in obtaining health care and preventive services [1].

It is estimated that over 15% of the world’s population—with up to 190 million (3.8%) of this specific demographic who are aged 15 and older experiencing serious functional challenges—frequently require health care facilities [2]. On the basis of household surveys carried out on child functional status, the United Nations International Children’s Emergency Fund (UNICEF) estimated that 28.9 million (4.3%) 0–4-year-olds, 207.4 million (12.5%) 5–17-year-olds, and 236.4 million (10%) 0–17-year-olds had moderate-to-severe impairments [3]. On the other hand, the Global Burden of Disease (GBD) 2019 reported estimates of 49.8 million (7.5%) children who were less than 5 years old, 241.5 million (12.6%) children between 5–19 years, and 291.3 million (11.3%) children younger than 20 years old to all have mild-to-severe impairments [4].

Common oral disease, such as dental caries, are known to afflict children; however, CSHCN possess a greater incidence rate of this disease than the general population [5,6,7]. This can be observed in children with limitations, such as impaired cognitive abilities, behavioural problems, impaired mobility, neuromuscular problems (drooling, gagging, and swallowing problems), uncontrolled body movements, gastroesophageal reflux, or seizures, where these complications can lead to inadequate oral care and an increased risk of developing oral health issues. Among the risk factors for oral illnesses in this demographic are their own oral ailments, since some genetic abnormalities in early children may cause deficiencies in tooth enamel, difficulties brushing, decreased salivary flow, medicines, and limited diets [8].

The implementation of efforts to improve oral health in order to reduce oral health inequality in children requires a deeper understanding of the intermediate processes that account for the large socioeconomic gradient in oral health in the CSHCN population; further, by conducting this, the impact of caregivers on the dental caries status of this group is highlighted. Moreover, it is well acknowledged that parents play a pivotal role in oral health promotion programmes that are designed to prevent dental caries in the CSHCN [9,10].

### Rationale of Study and Objective

Family is recognised as a significant factor in regard to health, it may be considered the main factor in the case of special needs people, since they may be completely dependent on their families for their basic needs [11]. As parents are the primary social force influencing child development in the early childhood years, it follows that interventions targeting parental beliefs and practices known to be associated with dental caries may be beneficial, especially among the CSHCN population. Therefore, the purpose of this review is to examine family influences, such as family traits, social determinants, parental attitudes, and oral hygiene behaviours on the dental caries status of the CSHCN population.

## 2. Materials and Methods

### 2.1. Systematic Literature Search

This review followed the PRISMA (preferred reporting items for systematic reviews and meta-analyses) standards [12,13].

#### 2.1.1. Inclusion Criteria

In order to be eligible, articles were required to meet the following criteria:

- Published in a peer-reviewed journal.
- Focused primarily on CSHCN out of any other disability group, as defined in the background section.
- Include the prevalence OR association/correlation between family influences variables and dental caries status of the CSHCN.
- Any cross-sectional study that involved family influences, including parental influences, parent–parent relationship, parent–child relationship, oral health behaviours performed on the child, and parental attitude (which, specifically, had an impact towards the dental caries status of the CSHCN).
- As CSHCN are usually dependent on their parents, the majority of the measurement tools used in the included studies were parental self-reports. These criteria are acceptable and papers that have used this method were also included. Most included studies also varied in the disability characteristics reported, thus, this variable was not evaluated in this review.

#### 2.1.2. Exclusion Criteria

- Papers published in languages other than English.
- Papers that included populations other than CSHCN.
- Papers that included an age group higher than 18 years old.

### 2.2. Information Sources

Electronic searches were conducted in the PubMed, Ebsco, Web of Science (WOS), Scopus, and Embase databases. Each database was searched by the author (DA) in order to perform an initial screening for relevancy, using study titles and abstracts. Another co-author (MA) performed the same method of searching in order to confirm the consistency in the number of outcomes obtained from these databases. Next, a random sample of abstracts was assessed by two additional co-authors (YY and BS). The intra-rater reliability test resulted in a Kappa value of 0.70, indicating a high agreement in regard to the inclusion of abstracts in the research. Further, a consensus was reached among the authors in the case of any disagreements. The search run was conducted from June 2022 until August 2022.

### 2.3. Search Strategy

The subject heading and medical subject heading (MeSH) terms that were used were combined, re-adapted, and re-adjusted accordingly in each database. These terms all included the following terms:

(1) (Family functioning or family function or family dysfunction) OR family relationships OR family structure OR family influence OR family characteristics OR parental influence;
(2) children with disabilities OR disabled children OR handicapped children;
(3) dental decay OR carious lesion OR carious dentin OR dental white spot OR dental caries OR (dental caries or dental decay or dental cavity or dental cavities or tooth decay).

### 2.4. Selection Process

**Figure A1:**
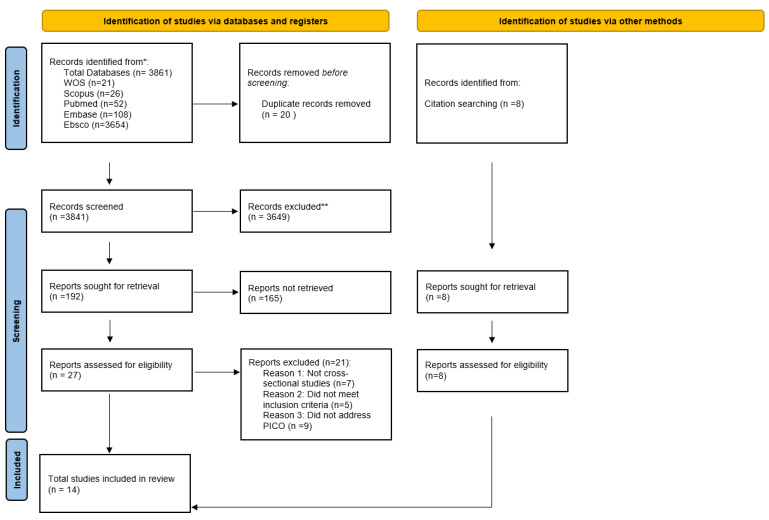
PRISMA flowchart to show the selection of articles.

The flowchart below illustrates the procedures and approach used to determine the qualifying articles (Figure A1).

The database search produced 3861 results (21 from WOS, 26 from Scopus, 52 from PubMed, 108 from Embase, and 3654 from Ebsco), of which 20 were duplicates. Through the screening of titles and abstracts, 3649 out of a total of 3841 references were eliminated. The remaining 192 references’ full-text articles were evaluated for eligibility. After reviewing the publications, six that met the inclusion criteria were found. Eight studies that met the inclusion criteria were found via a hand search. In total, 14 studies were considered in this analysis for final review. All of these 14 investigations were performed from 2010 to 2021. All of the studies included in this evaluation used cross-sectional designs, and no temporal constraints were imposed on the search.

Studies were omitted if they: were reviews or editorials, utilised a sample older than 18 years, did not include CSHCN, did not measure dental caries, did not analyse familial/parental variables, or employed a research design other than cross-sectional. Studies that reported the prevalence by percentage were also considered. The main author (DA) of this study, extracted information independently using a checklist and tables. The principal author also used Excel in order to organise the screening procedure. All findings were independently examined twice, and any discrepancies were addressed by the reviewers’ consensus (YY and BS). The whole text of every study was reviewed and any remaining incomplete documents were located and also reviewed. The main author manually hand-searched the reference lists of all included studies in order to identify the primary sources and significant measurement developments and/or validation references; in addition, citation tracking was also performed on these papers. Disputes were resolved by reviewing documents and engaging in a discussion until a consensus was achieved.

### 2.5. Quality Assessment

The National Heart, Lung, and Blood Institute quality assessment tool for cross-sectional study was used to assess the risk of bias in all articles (Table A1). There were 14 items listed in the assessment tool, however, 4 of the items (9, 10, 12, 13) that assessed the cohort study properties were excluded and considered as ‘not applicable’ to the intended type of study. Items marked as ‘Yes’ were given a score of 1 each. The total score for all items was then calculated and labelled according to these categories: ‘Good’ (if they fulfilled 60–100% of the tool items); ‘Fair’ (if they fulfilled 50–59% of the tool items); or ‘Poor’ (if they fulfilled 0–49% of the tool items).

### 2.6. Dealing with Lack of Information

Where more information was unavailable in the primary reference, supplementary publications were sought for. If information remained unavailable, it was noted as “not reported”.

### 2.7. Method of Analysis and Synthesis

The data were synthesized in a narrative fashion and presented in structured table with the primary outcomes. The aim here was to provide an overview of the variables, including the measurements used for both family influence and dental caries variables.

## 3. Results

### 3.1. Measurement Tool for Family Influence

The list of measurement tools used by the reviewed studies during reporting the family influences varied. The tools included: the Brazilian Early Childhood Oral Health Impact Scale (B-ECOHIS); the Patient Health Questionnaire (PHQ): PHQ-2 and PHQ-9; the Alcohol Use Disorders Identification Test (AUDIT); the Fagerstrom test for nicotine dependence (FTND); the Parental–Caregivers Perception Questionnaire (P–CPQ); the World Health Organization Quality of Life–BREF (WHOQOL–BREF); and the Child Perception Questionnaire (CPQ). The purpose of each measurement tool is presented below:

- B-ECOHIS: Used in order to evaluate the detrimental effects of caries on the quality of life of CSHCN [14].
- PHQ-2: A questionnaire measuring carers’ prevalent depressive symptoms [15], and the frequency and severity of depressed symptoms during the previous two weeks from when the study took place [15].
- PHQ-9: Measures the frequency and severity of depressed symptoms during the previous two weeks from when the study took place [16].
- WHOQOL-BREF: A questionnaire that addresses physical, psychological, social, and environmental domains [17].
- AUDIT: This test consists of ten questions on the last twelve months of hazardous and detrimental alcohol use [18].
- FTND: This test is administered to all caregivers to identify nicotine dependency [19].
- P–CPQ: Examines the parental assessments of the OHRQOL of their children [20].
- CPQ: A self-report questionnaire that is given to the caregivers in order to assess the impact of their child’s current oral health on their everyday life [21].

### 3.2. Reliability and Validity

Only one study reported on the reliability of the measurement used [22]. The highest–retest reliability (>0.70) was noted for all variables in the questionnaire reported by the parent/carer and the results of the oral examination of the kids. Five other studies failed to report on the reliability and validity scores [23,24,25,26,27].

### 3.3. Measurement Tool for Dental Caries

#### DMFT/dmft

This index provides a person’s or group’s caries history. This index provides a fast view on how the tooth has been damaged by caries, but it does not provide a preventative view since it does not identify lesions in their earliest stages. It is unable to identify the existence of dental caries lesions, hence limiting the capacity to comprehend the genuine oral health condition surrounding dental caries. The DMFT index assessment is performed on 28 permanent teeth (omitting the third molars), or on the 32 permanent teeth as well [28]. Dmft, on the other hand, is used with the same purpose, but in the primary teeth.

These indices are numerical measures of the caries prevalence of an individual, or group, and are often used in oral health epidemiological studies. It is determined by totalling the number of permanent teeth impacted by caries, where D/d represents decay, M/m represents caries-related tooth loss, and F represents filled teeth (T). If a tooth has both a filling and a caries lesion, it is counted as D/d in the DMFT/dmft index.

All of the studies included in this review used this index in order to represent the dental caries prevalence and its association/correlation with family influence variables.

### 3.4. Risk of Bias within Studies

The methodological quality scores for this review range from 0–10 and is reported in a percentage in order to indicate whether the particular study can be characterized as “Good”, “Fair”, or “Poor”. Ten out of the fourteen included cross-sectional studies scored “Good”, thus were considered high-quality studies. Consequently, due to the small number of articles found in this review, papers marked as fair/poor were also included during the reporting of the findings. Information regarding the quality of the individual studies is provided in Appendix A.

### 3.5. Characteristics of Study

The majority of the studies were conducted in Asia, which include Taiwan, Hong Kong, China, Bangladesh, India, and Indonesia [22,24,25,26,27,29,30,31]. Three other studies were conducted in Brazil [32,33,34], and the remaining two were conducted in Saudi Arabia and South Africa [23,35]. The distribution of age across the studies approximated a normal distribution, and all conformed to a definition of “children” that ranged between 0–18 years old [36].

A summary of the parental variables that were explored are presented in Table 1. These have been divided into the following categories: socio-demographic (age, sample size, and type of disability); measurement index for family influence variables; and main outcomes (caries prevalence, oral health-related behaviours, parents’ education level, parents’ occupation level, frequency of sugar taking, dental visit, parents’ tobacco/alcohol use, and the parents’ depression status and its association). The details of each variable outcome are profiled below:

### 3.6. Findings

#### 3.6.1. Prevalence of Caries

Most of the studies had a caries prevalence of over 50% [22,23,24,25,26,29,37], with the highest being 100% of the children being presented with caries [30]. The lowest prevalence of caries was found in Ni Zhou et al., which documented only 30.3% of the children affected by the disease. Only two studies failed to report the overall prevalence of caries [33,34].

#### 3.6.2. Employment and Economic Status

Lower socioeconomic class and family income are often related with a greater frequency of dental caries; nevertheless, all investigations found null results. Family employment, such as parents’ occupation levels, showed that the children of parents who both possessed high-skilled levels of occupation had significantly lower DMFT indices and caries prevalence than the children of the parents who both possessed unskilled levels [24].

#### 3.6.3. Family Size and Birth Order

Family size and birth order may affect the amount of the resources available to siblings and have been linked to children’s health outcomes [38]. Although there was no study in this review that looked at the higher birth order, one study has shown that there is a significantly higher number of dental caries in the CSHCN with families consisting of a large family size (i.e., four or more people) although this finding was not universal [34].

#### 3.6.4. Education

Education level is a significant socioeconomic indicator that represents the information and skills necessary in order to make healthy lifestyle decisions [39]. For instance, more educated parents express more favourable views and greater intentions to limit their children’s sugar consumption than less educated parents [10]. One study has shown a significant correlation between education level of parents with dental caries, where parents with both high education level would have lesser dt + DT, deft + DMFT indices, and caries prevalence than children of the parents with lower education level [24].

#### 3.6.5. Tooth Brushing

Five studies in this review have included the frequency of toothbrushing, and assistance in toothbrushing, as one of their outcome variables. There is also some contention regarding the association between tooth brushing behaviours and dental caries. Three of the studies have shown higher statistically significant caries prevalence among children who brushed their teeth by themselves than those children whose teeth were brushed by their parents/caregivers [24,27,34]. However, two of the studies have reported otherwise, where no association was observed between brushing assistance and dental caries [25,26].

Frequency of brushing was found to be associated with dental caries in one study [27], but not significantly associated in Jawed et al. (*p* = 0.139) [26]. Interestingly, one finding showed that children who did not practice brushing teeth after snacking and stopped brushing early had significantly higher caries than those who did [22].

#### 3.6.6. Dental Attendance

In this review, two studies have shown that there is a significant association between dental services and dental caries with one study [25] specifically looking at the number of dentist visits (*p* < 0.05) and the other at the reason for visiting the dentist (e.g., only visited a dentist for a certain problem) (*p* = 0.02) [22].

#### 3.6.7. Parental Oral Health

An association between the dental indices of children and their parents is shown in [40]. One research [34] also found that parents with a history of dental issues would result in their children being more susceptible to developing caries.

#### 3.6.8. Parental Attributes

Parental attributes are features of the parent or caregiver that may impact the child’s development environment, such as the age and psychological states of the mother, for example. The influence of the mothers’ age was investigated in Barros et al. [34] and was seen to report a higher number of children with dmft > 0, especially within the range of 18–34 years old. A psychological variable/domain such as depression, however, was reported differently, that is: (1) In two studies it was reported that mothers who experienced worse conditions in the physical and psychological domains, included in 18–43 age group, showed a higher number of children with dmft > 0 (25.6%) [34,37]; (2) one study [33] showed no statistically significant associations with the caries index for depression and alcohol abuse (*p* = 0.276). Contrastingly, in the same study as well, it was reported that there is a significant positive correlation between dental treatment needs and caregivers’ tobacco use (*p* = 0.009) [33]. Garate et al. [33] also reported that there was no statistically significant association between tobacco use and alcohol with dental caries (*p* > 0.05).

#### 3.6.9. Parental Attitudes, Knowledge, and Beliefs

Parental attitudes, knowledge, and beliefs impact the decisions parents make for their children, the behaviours they model for their children, and the tastes and preferences children acquire throughout childhood [41]. Children who actively asked for sweets and often received sweets from parents, caregivers, or schoolteachers as a reward for behaviour control, showed statistically significantly higher dt + DT and caries prevalence (64.74%, *p* = 0.0155) compared to those who did not [27]. Children who snack twice a day or more showed a higher caries outcome and had a significant association with dental caries (*p* = 0.02) [22]. However, Barros et al. [34] has shown no positive correlation or significant association between parents’ information on prevention of dental caries with the dental caries status itself (*p* > 0.05).

## 4. Discussion

Most of the studies had a caries prevalence of over 50% [22,23,24,25,26,29,37], with the highest being 100% of the children being presented with caries [30]. The significance of risk factors, such as the psychological states of the parents and their behaviour toward their children in influencing the dental caries status of CSHCN is an extremely valuable finding of this review. Although, it must be noted that other findings, such as the frequency of brushing and snacking habits of CSHCN, were inconsistent.

### 4.1. Quality of Study

Due to the restricted number of final articles acquired after applying the selection criteria, papers that rated poor/fair were also included in order to diversify the results of this study, as stated earlier. The absence of inclusion/exclusion criteria and the ineffectiveness of sample size computation were the primary contributors to the low quality of the publications. Therefore, this evaluation should not be regarded as conclusive, and other research must be conducted in order to provide more concrete data, particularly regarding the association of certain family traits with the dental caries status of the CSHCN.

### 4.2. Caries Prevalence

In general, the prevalence of caries in CSHCN is seen to be higher than in non-CSHCN, due to several limitations faced by the CSHCN population. Similar data are reported in previous research, indicating that CSHCN have more instances of decaying and missing teeth than in other populations. These discrepancies may be attributable to the cumulative neglect of dental health in children with special needs, and the severe state of untreated caries that necessitates extraction [42,43]. In addition, poor oral health and a high risk fraction of untreated caries in children with special needs may be a result of a lack of cooperation, which makes it difficult to provide dental care and attend dental services, as shown in two studies in this review, i.e., Gadiyar et al. [25] and Hariyani et al. [22].

### 4.3. Socio-Demographic Status

Certainly, socioeconomic position (such as a lower education level and the economic standing of their parents), will have a significant influence on the quality in the oral health status of children who are with impairments [44]. In our study, the dental health status and caries prevalence of the CSHCN were significantly related to their parents’ education and occupation level, respectively.

The number of people per family was also shown to influence the dental caries occurrence in this population, as reported in Barros et al. [34], which is also consistent with another study [45]. This may be due to the parents needing to share their attention with other people or to cater with their feelings of isolation, in addition to caring for a child with a disability, which may restrict their time and, subsequently, their ability to care for the child’s oral health.

### 4.4. Tooth Brushing

As the majority of children with disabilities—particularly those with severe or profound impairments in cognition, memory, communication, and physical dexterity—are unable to fully care for themselves in day-to-day activities, there is a surge in their need for specialised equipment. The same situation happens in the tooth-brushing behaviour of those children with disabilities. As they cannot control their own diet, whether they have sweetened meals, or if they clean their teeth correctly, their parents or caretakers are seen to be mostly responsible for these daily dental care chores. As it is difficult to achieve proper brushing, especially in regard to CSHCN, they may only be able to achieve adequate oral hygiene by their teeth being brushed more than once each day with the support of their parents or carers, thus, imposing a lower caries prevalence, as shown in the outcomes of Liu et al. [29], Hsiao et al. [27], and Barros et al. [34]. This finding is also consistent with the previous study [46].

### 4.5. Snacking Habit

In regard to snacking habits, the CSHCN who snacked at least twice daily were more likely to have severe caries than those who snacked seldomly, as reported in Hsiao et al. [27] and Hariyani et al. [22]. These situations, when repeated every day, developed habits that made children’s caries progressively worse. These results are consistent with the findings of Savage et al. in their research on parental attitudes, which demonstrated that parents’ behaviour influences their children’s development in regard to healthy eating habits [47]. Food is one of the most often utilised “rewards” used in order to control behaviour. Traditionally, behaviour modification programmes for children with behavioural problems or mental disabilities, have employed candy, gum, carbonated drinks, and sweetened juices as a reward due to their cheap cost and high desirability [48]. This will, therefore, develop a preference towards unhealthy snacking in time among the CSHCN, who are usually are dependent on their parents/caregivers.

### 4.6. Psychological Domain

Psychological variables, such as depression, were associated with higher levels of caries in children, as reported by Garate et al. [33], Akhter et al. [37], and Barros et al. [34]. In addition, mothers of disabled children may have less free time, a greater change in their professional lives, and a greater sense of isolation, all of which can interfere with their personal and social relationships and cause them to be more stressed than mothers of healthy children, who did not have to restructure their entire lifestyles to provide care, thereby depriving them of personal and social relationships [49,50].

A higher incidence of dental caries in the children of smokers may be one of a number of negative health consequences for them, however, in development of this review, it was found that Garate et al. [33] demonstrated that there is no significant association between tobacco use and alcohol consumption in regard to dental caries. Recently, a study has shown that there is a positive association between tobacco smoking and dental caries [51]. This scarcity in data is probably due to the limited studies that have specifically looked at CSHCN as the target population.

### 4.7. Parental Knowledge and Belief

Barros et al. [34] also found that there was no association between dental caries and the prevention information obtained by parents. The explanation for this may be due to the caretakers’ view that the severity of the disability is seen to have a greater impact on the overall health of the child rather than the oral health-related issues overall. This demonstrates the lack of understanding among caregivers about the significance of dental health to overall health. Most demographic groups do not regard oral health to be a life-threatening condition [52]. Providing caregivers of CSHCN with information is crucial in order to empower them to cope with their children’s impairment and assist with their daily care. Families must realise that preventative measures, such as daily brushing and frequent dental appointments, avert more complicated circumstances.

## 5. Suggestions

Improving parental awareness and education may continue to be the most important factor to consider when attempting to lower the incidence of caries in children. Information is crucial for the purposes of enabling parents of CSHCN to manage their children’s impairment and assist in their daily care. Families must realise that preventive measures, such as daily tooth brushing and regular dental exams, avert more threatening complications. Dentists play the most crucial role in ensuring the success of this initiative in order to empower families, as well as encourage self-care. There will be an urgent need to reach agreement on clear, concise, and evidence-based oral health recommendations, both within the dentistry profession and across other disciplines that may have a role in children’s oral health, e.g., by implementing guidelines broadly. While brushing with fluoride-containing toothpaste is considered the easiest technique to prevent tooth decay, there are other measures that may be taken, such as through the usage of a recent SDF method in targeting high-risk patients. Finally, lower income SES families face economic difficulties, which have a negative impact on family food security and dietary quality. Hence, policies that provide food assistance and subsidies toward families with CSHCN must be emphasized, as is also suggested in these studies [53,54].

## 6. Strength

There are few studies on parental attitudes, knowledge, and beliefs, particularly among the CSHCN population, despite the fact that demographic characteristics are often studied in relation to dental caries. Psychologists with expertise in the study of attitudes, knowledge, beliefs, in the development and maintenance of behaviours, and parenting and parent–child interactions can play a crucial role in elucidating the motivators and maintenance behaviours of parents when they make health-related decisions for their children. Although the search has revealed only a few related papers that could be included in this study, it can still act as a jumpstart to explore the family influences that affect the CSHCN population particularly.

## 7. Limitations

### 7.1. Reporting Bias

A small proportion of parents or caregivers may respond to questions so as to match societal expectations, resulting in response bias.

### 7.2. Nature of Study

This research is limited by its cross-sectional methodology, which does not provide the validation of a causal hypothesis. Longitudinal and prospective multifactorial studies are required in order to assess the effect of sociocultural variables, parental factors, and child behaviours on the onset of dental caries. Lastly, the cross-sectional design of the majority of research gives a picture of characteristics that are linked with dental caries, but not causal pathways.

## 8. Conclusions

Family influences—which include tooth brushing behaviour, parents’ education level, parents’ occupation level, diet pattern, dental visit, snacking frequency, parents’ psychological status, family size, and parents’ knowledge towards oral health—imparted a significant relationship and association towards the dental caries status of the CSHCN population.

The results obtained highlight the importance of introducing preventive programs for better control of dental caries. The development and strengthening of governmental policies for the inclusion of children with disabilities in schools and cultural contexts may have a dual beneficial effect. On the one hand, the immediate socio-educational and cultural advantage to the child, and on the other, more space and time for the mother to engage in more social engagement and integration, which will undoubtedly have an effect on the psychological domain.

## 9. Other Information

This review has been registered in PROSPERO with registration number CRD42021274923.

## Figures and Tables

**Table 1:** Summary of included studies.

Author, Year, Country	Sample Characteristics			Measurement		Main Outcomes	Risk Assessment Score
	Age	Sample Size	Type of Disability	Family Influence	Dental Caries		
[23]; Saudi Arabia	6–18 years	75	Autistic Spectrum Disorder (ASD)	Self-developed (Self-dev), pilot tested Not tested for validity and reliability	dmft, DMFT	Caries prevalence; primary teeth (76%), permanent teeth (68%)	Good
[32]; Brazil	1–9 years	128	Multiple	FIS	dmft, DMFT	Caries prevalence; (44.5%)	Good
[24]; Taiwan	6–12 years	535	Multiple	Self-administered (self-ad) questionnaire, adapted Validated but not reported	dmft, DMFT	Caries prevalence; (58.69%) Children who brushed their teeth by themselves had statistically significantly lower caries prevalence (35.216%) than those children who brushed teeth by themselves. Parents with both high education level would have lesser dt + DT, deft + DMFT indices and caries prevalence than children of the parents with lower education level. Parents’ occupation levels also showed that the children of parents with both high skilled levels had lower, and significantly lower dt + DT, deft + DMFT indices and caries prevalence (*p* = 0.0061) than the children of the parents with both unskilled levels.	Good
[25]; India	Mean age: 13.85 years	223	N/M	Self-dev questionnaire, pilot tested Validity and reliability are not reported	DMFT	Caries prevalence; (68.6%) Statistically significant association between dental visit and brushing frequency with dental caries. No significant association between brushing assistance + sugar consumption (with meals and in between meals) with dental caries.	Poor
[33]; Brazil	3–18 years	151	Medical condition (MD), Intellectual disability (ID)	PHQ, AUDIT, FTND	DMFT	No statistically significant association between tobacco use and alcohol with dental caries. No statistically significant association between depression and alcohol abuse. Significant positive correlation between dental treatment needs and caregivers’ tobacco use.	Fair
[26]; Pakistan	6–18 years	196	deaf, ID, down syndrome (DS), ASD, vision, cerebral palsy (CP)	Self-ad questionnaire adapted from other study. Validity and reliability are not reported.	dmft, DMFT	Caries prevalence; (58.2%) No significant association frequency of tooth brushing and the presence of dental caries. No significant association was found between dental caries status and supervised tooth brushing.	Good
[35]; South Africa	Mean age: 8.7 ± 6.07 years	150	CP, hydrocephalus, ASD, epilepsy, and Global developmental delays (GDD)	P-CPQ	dmft, DMFT	Caries prevalence; (42%) The number of teeth affected by dental caries in the primary dentition was significantly correlated with oral symptoms, functional limitations, and social well-being domains.	Good
[30]; Indonesia	N/M	40	N/R	ECOHIS, SOHO- 5, OHRQoL-C5, COIDP, COHRQoL, P-CPQ	dft, DMFT	Caries prevalence; 100%	Poor
[27]; Taiwan	6–12 years	484	Multiple	Self-reported questionnaire. Validity and reliability are not reported.	ds + Ds	Children who asked for sweets, frequently had sweets, had independent tooth-brushing abilities, or infrequently brushed their teeth every day tended to have statistically significantly higher decayed teeth.	Fair
[22]; Indonesia	≤12 years old and ≥13 years	70	ASD	Self-ad questionnaire. High test–retest reliability (>0.70) was noted for all variables in the questionnaire reported by the parent- carer and the results of the oral examination of the kids.	dft, DMFT	Caries prevalence; (78.6%) Children who finished brushing due to kids rejection, had snacking frequency twice a day or more, did nothing after eating snack and only visited a dentist for a problem, have significantly higher caries both measured as DMF-T/dmf-t compared to who finished brushing due to carer’s clean standard, had limited snacking frequency, had a habit of drinking water or brushing the teeth after eating snack, and visited a dentist for a check-up, respectively. Snacking frequency was significantly associated with increased number of teeth experiencing caries.	Good
[34]; Brazil	2–6 years	67	Multiple	WHOQOL–BREF, self-ad questionnaire, Multidimensional Health Locus of Control Scale	dft	Mothers who reported worse conditions of physical and psychological domains, included in 18-43 age group showed a higher number of children with dmft > 0 (25.6%). Families consisting of four or more people have more children with dmft index > 0 (31.4%) when compared to families with fewer people. Children who brush their teeth by themselves (33.3%) have higher dmft index, compared with those who receive help from an adult to do it (23.9%). 96% of children had already seen a dentist, who gave their mothers information on prevention of oral diseases, although 23.4% of the children had presented dmft > 0 index.	Good
[31]; Hong Kong	2–6 years	383	Multiple	Self-ad questionnaire. Validity and reliability are not reported.	dmfs	Caries prevalence; (30.3%)	Good
[37]; Bangladesh	2–17 years	90	CP	CPQ, FIS	dmft, DMFT	Caries prevalence; (52.2%) Significant associations with dental caries experience and FIS parents reports of ‘felt frequently guilty’ (*p* = 0.001) and ‘being upset’ (*p* = 0.0001) Dental caries experience was significantly associated with CPQ and FIS scores among children and adolescents with CP; especially in those children and adolescents who reported feeling upset frequently (*p* = 0.02).	Good
[29]; China	12–17 years	450	Multiple	Self-ad questionnaire. Validity and reliability are not reported.	DMFT	Caries prevalence; (53.5%)	Good

N/M: Not mentioned; N/R: Not reported.

## Data Availability

Not applicable.

## Appendix A

**Table A1:** NHLBI quality assessment tool used for the observational cohort and cross-sectional studies.

Item	[23]	[32]	[24]	[25]	[33]	[26]	[35]	[30]	[27]	[22]	[34]	[31]	[37]	[29]
1. Was the research question or objective in this paper clearly stated?	Y	Y	Y	Y	Y	Y	Y	N	Y	Y	Y	Y	Y	Y
2. Was the study population clearly specified and defined?	Y	Y	Y	N	Y	Y	Y	Y	Y	Y	Y	Y	Y	Y
3. Was the participation rate of eligible persons at least 50%?	Y	Y	Y	Y	NR	Y	N	NR	NR	Y	Y	Y	Y	Y
4. Were all the subjects selected or recruited from the same or similar populations (including the same time)? Were the inclusion and exclusion criteria in the study prespecified and applied uniformly to all participants?	Y	Y	Y	N	N	Y	N	N	N	N	Y	N	Y	Y
5. Were sample size justification, power description, or variance and effect estimates provided?	N	Y	N	N	N	Y	Y	N	N	N	N	Y	NR	N
6. For the analyses in this paper, were the exposure(s) of interest measured prior to the outcome(s) being measured?	N	N	N	N	N	N	N	N	N	Y	N	N	Y	Y
7. Was the timeframe sufficient such that one could reasonably expect to see an association between exposure and outcome if it existed?	N/A	N/A	N/A	N/A	N/A	N/A	N/A	N/A	N/A	N/A	N/A	N/A	N/A	N/A
8. For exposures that can vary in amount or level, did the study examine different levels of the exposure as related to the outcome (e.g., categories of exposure or exposure measured as continuous variable)?	N/A	N/A	N/A	N/A	N/A	N/A	N/A	N/A	N/A	N/A	N/A	N/A	N/A	N/A
9. Were the exposure measures (independent variables) clearly defined, valid, reliable, and implemented consistently across all study participants?	Y	Y	Y	Y	Y	Y	Y	Y	Y	Y	Y	Y	Y	Y
10. Was the exposure(s) assessed more than once over time?	N	N	N	N	N	N	N	N	N	Y	N	N	N	N
11. Were the outcome measures (dependent variables) clearly defined, valid, reliable, and implemented consistently across all study participants?	Y	Y	Y	Y	Y	Y	Y	Y	Y	Y	Y	Y	Y	Y
12. Were the outcome assessors blinded to the exposure status of participants?	N/A	N/A	N/A	N/A	N/A	N/A	N/A	N/A	N/A	N/A	N/A	N/A	N/A	N/A
13. Was the loss to follow-up after baseline 20% or less?	N/A	N/A	N/A	N/A	N/A	N/A	N/A	N/A	N/A	N/A	N/A	N/A	N/A	N/A
14. Were key potential confounding variables measured and adjusted statistically for their impact on the relationship between exposure(s) and outcome(s)?	Y	Y	Y	N	Y	Y	Y	Y	Y	Y	Y	Y	Y	Y
Risk assessment in % (Good/Fair/Poor)	70% (Good)	80% (Good)	70% (Good)	40% (Poor)	50% (Fair)	80% (Good)	60% (Good)	40% (Poor)	50% (Fair)	80% (Good)	70% (Good)	70% (Good)	80% (Good)	80% (Good)

Good if they fulfilled 60–100% of the tool items, fair if 50–59%, or poor if 0–49%. Y = Yes, N = no, CD = cannot determine, NR = not reported, and NA = not applicable.
